# P300/CBP-Associated Factor Activates Cardiac Fibroblasts by SMAD2 Acetylation

**DOI:** 10.3390/ijms22189944

**Published:** 2021-09-14

**Authors:** Yongwoon Lim, Anna Jeong, Duk-Hwa Kwon, Yeong-Un Lee, Young-Kook Kim, Youngkeun Ahn, Taewon Kook, Woo-Jin Park, Hyun Kook

**Affiliations:** 1 Department of Pharmacology, Chonnam National University Medical School, Hwasun 58128, Jeollanamdo, Korea; dyddns2216@gmail.com (Y.L.); annaj0330@gmail.com (A.J.); elio9359@daum.net (D.-H.K.); gloryw3@korea.kr (Y.-U.L.); twkook1020@gm.gist.ac.kr (T.K.); 2 Basic Research Laboratory for Vascular Remodeling, Chonnam National University Medical School, Hwasun 58128, Jeollanamdo, Korea; ykk@jnu.ac.kr (Y.-K.K.); cecilyk@chonnam.ac.kr (Y.A.); 3 BK21 Plus Center for Creative Biomedical Scientists, Chonnam National University, Gwangju 61469, Korea; 4 BioMedical Sciences Graduate Program (BMSGP), Chonnam National University, Hwasun 58128, Korea; 5 Health and Environment Research Institute of Gwangju, 584, Mujin-daero, Seo-gu, Gwangju 61954, Korea; 6 Department of Biochemistry, Chonnam National University Medical School, Hwasun 58128, Jeollanamdo, Korea; 7 Department of Cardiology, Chonnam National University Hospital, Gwangju 61469, Korea; 8 College of Life Sciences, Gwangju Institute of Science and Technology, Gwangju 61005, Korea; wjpark@gist.ac.kr

**Keywords:** cardiac fibrosis, cardiac fibroblasts, P300/CBP-associated factor, transforming growth factor-β1, acetylation, posttranslational modification

## Abstract

Various heart diseases cause cardiac remodeling, which in turn leads to ineffective contraction. Although it is an adaptive response to injury, cardiac fibrosis contributes to this remodeling, for which the reactivation of quiescent myofibroblasts is a key feature. In the present study, we investigated the role of the p300/CBP-associated factor (PCAF), a histone acetyltransferase, in the activation of cardiac fibroblasts. An intraperitoneal (i.p.) injection of a high dose (160 mg/kg) of isoproterenol (ISP) induced cardiac fibrosis and reduced the amount of the PCAF in cardiac fibroblasts in the mouse heart. However, the PCAF activity was significantly increased in cardiac fibroblasts, but not in cardiomyocytes, obtained from ISP-administered mice. An in vitro study using human cardiac fibroblast cells recapitulated the in vivo results; an treatment with transforming growth factor-β1 (TGF-β1) reduced the PCAF, whereas it activated the PCAF in the fibroblasts. *PCAF siRNA* attenuated the TGF-β1-induced increase in and translocation of fibrosis marker proteins. *PCAF siRNA* blocked TGF-β1-mediated gel contraction and cell migration. The PCAF directly interacted with and acetylated mothers against decapentaplegic homolog 2 (SMAD2). *PCAF siRNA* prevented TGF-β1-induced phosphorylation and the nuclear localization of SMAD2. These results suggest that the increase in PCAF activity during cardiac fibrosis may participate in SMAD2 acetylation and thereby in its activation.

## 1. Introduction

Heart diseases are the leading cause of morbidity and mortality worldwide and account for nearly a third of deaths in resource-rich countries. Heart failure is a clinical syndrome defined as the inability of the heart to adequately meet the demands of the body for blood and oxygen. Thus, it is considered as the final path of various heart diseases. The progression of cardiac remodeling is closely related to structural and functional impairment in the heart under diverse conditions, such as hemodynamic stresses or cardiac injury. Either physiologic or pathologic stresses can cause cardiac remodeling; while physiologic cardiac remodeling is an adaptive response to an increase in load and demands, pathologic remodeling is a maladaptive response to pathologic stimuli such as ischemia/reperfusion and overload, involving myocyte death, inflammation, and diffuse fibrosis [1,2]. Cardiac fibrosis is a scarring event after cardiac injury, characterized by the proliferation of fibroblasts and the depositing of the extracellular matrix (ECM), but is also considered as a key contributor to pathologic remodeling and the progression to heart failure [3]. Indeed, prolonged and advanced fibrosis in the heart is strongly associated with poor clinical outcomes, including mortality, atrial fibrillation, and heart failure [4,5]. Despite substantial advances in therapeutic strategies, effective antifibrotic therapies remain unavailable [3]. Therefore, we need to better understand the fundamental mechanisms driving cardiac fibrosis to develop specific effective therapeutics.

The heart is composed of multiple cell types, including myocytes, fibroblasts, and endothelial cells. Cardiac remolding is a complex process that involves a combination of cellular and molecular alterations under neurohumoral activation and hemodynamic load. During cardiac remodeling, myocytes, the most abundant cardiac cells, undergo changes in their size, shape, and contractile activity; re-expression of the fetal gene program; apoptosis; and necrosis [6]. Resident quiescent fibroblasts begin to proliferate and differentiate, which leads to cardiac fibrosis. Persistent and progressive cardiac fibrosis results in an increase in cardiac stiffness and abnormalities in matrix composition and quality, which eventually causes cardiac dysfunction [7,8]. Recent studies have shown that nonmyocyte cells, including pericytes, bone-marrow-derived progenitor cells, and vascular smooth muscle cells, can differentiate into myofibroblasts through phenotypic switching after mechanical stresses [9,10]. Additionally, both epithelial and endothelial cells are also involved in the process of fibrosis through sequences known as the epithelial-to-mesenchymal transition (EMT) and endothelial-to-mesenchymal transition (EndMT), respectively [11,12].

Various cytokines and signaling pathways have been identified to be involved in the development of cardiac fibrosis. Among them, the TGF-β superfamily is a well-known profibrotic cytokine secreted by different cell types. The TGF-β family comprises 33 members and participates in diverse cellular processes, such as cell fate determination, the cell cycle, and cell migration [13]. Mechanistically, TGF-β1, a key molecule in fibrogenesis, binds to receptors and induces phosphorylation of regulatory SMADs, including SMAD2 and SMAD3. Phosphorylated SMAD2 and SMAD3 then directly interact with their common mediator SMAD4 and are then incorporated into the nucleus to activate transcriptional expression of downstream genes, such as alpha-smooth muscle actin (*ACTA2*). In addition, several studies have demonstrated that TGF-β1 accelerates the development of fibrosis in a SMAD-independent manner, such as via mitogen-activated protein kinase pathways, the Hippo pathway, and the Wnt/β-catenin signaling pathway [14].

The post-translational modification (PTM) of histone proteins, such as acetylation or methylation, is a key epigenetic regulation mechanism that plays a crucial role in the transcriptional program and the following biological behaviors of the cells. Histone modification is tightly regulated by a balance between multiple classes of enzymes termed writers, erasers, and readers of histone proteins. Writers that catalyze the transfer of acetyl groups to specific lysine residues on histones are called lysine (K) acetyltransferases. In contrast, erasers, which remove the acetyl group from lysine residues on histones, are termed histone deacetylases (HDACs). Readers, on which less research has been done, possess unique domains that recognize the specific PTM status of the histones and are recruited to specific covalent “marks” on histones, which then relay the signals from the histones to the other parts of the cells [15]. Although those modifiers were initially known to act on histones only, a variety of nonhistone substrates have been determined to be regulated by the same enzymes [16,17]. Importantly, many studies have been carried out to determine the roles of different types of epigenetic modification in the maintenance of homeostasis in the heart [18,19]. However, the role of epigenetic regulation in cardiovascular diseases remains unclear.

Our laboratory has investigated the epigenetic mechanism and established various key molecules implicated in cardiovascular diseases, including vascular calcification and cardiac hypertrophy [20,21,22,23,24]. We previously reported that histone deacetylase 2 (HDAC2) promotes the development of cardiac hypertrophy, and its activity is tightly regulated by casein kinase 2α1-mediated phosphorylation [20,21]. Furthermore, we demonstrated that PCAF acetylates lysine 75 on HDAC2 and thereby increases its deacetylase activity, which otherwise remains suppressed by histone deacetylase 5 (HDAC5) [24]. The PCAF is a member of the GCN5-related N-acetyltransferase (GNAT) family of protein acetyltransferases and is known to play an important role in diverse biological processes, such as transcriptional regulation, chromatin remodeling, metabolism, and the cell cycle. Emerging evidence indicates that the PCAF is strongly associated with development and various types of diseases including coronary heart disease [25]. However, what pathogenic role the PCAF may play in the development of cardiac fibrosis is still not well understood. Recently, using a nondiabetic unilateral ureteral obstruction model, Kim et al. [26] reported that renal tubulointerstitial fibrosis is attenuated by administration of garcinol, a PCAF inhibitor. Furthermore, previous research demonstrated that PCAF-mediated acetylation of SMAD2, a profibrotic mediator of TGF-β, is required for its activity in HEK293T cells [27,28]. Taken together, these findings lead us to hypothesize that the PCAF may play crucial roles in the development of cardiac fibrosis. Here, we investigated whether the PCAF is involved in the development of cardiac fibrosis and myofibroblast differentiation.

## 2. Results

### 2.1. Cardiac Fibrosis Induces PCAF Activity in Cardiac Fibroblasts In Vivo

**Figure 1:**
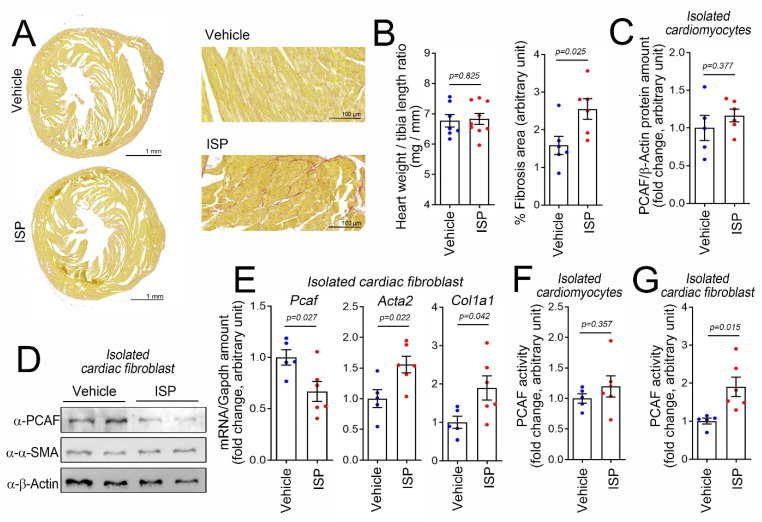
Intraperitoneal (i.p.) administration of high-dose isoproterenol (ISP) induced cardiac fibrotic stress and activates the p300/CBP-associated factor (PCAF) in cardiac fibroblasts in mice. (**A**) ISP (160 mg/kg, i.p.) injection caused fibrosis. Picrosirius red staining is shown. The mouse hearts were harvested 6 days after injection. Higher magnification images are provided in the panels on the right. (**B**) High-dose ISP did not induce enlargement of the heart but did increase the fibrosis area. (**C**) ISP did not alter the protein amount of the PCAF in the isolated cardiomyocytes obtained from the mouse heart. (**D**) ISP decreased the protein amounts of the PCAF in cardiac fibroblasts isolated from the mouse heart. α-SMA, a fibrosis marker protein, was slightly increased by ISP. (**E**) Changes in mRNA levels determined by quantitative RT-PCR. (**F**) PCAF acetylase activity in the isolated cardiomyocytes was not altered by ISP. (**G**) PCAF activity was significantly increased in the isolated cardiac fibroblasts by ISP. Each dot in the bar graphs shows the value of one case. Data are shown as mean ± standard error of the mean (SEM). *p*-values for the different comparisons are shown in the figure.

To investigate the role of the PCAF in cardiac fibrosis, we first induced cardiac fibrosis in vivo. A single, high-dose intraperitoneal (i.p.) injection of isoproterenol (ISP), a beta-adrenergic agonist, can induce cardiac fibrosis in mice in a relatively short period [29]. Using this model, we observed an increase in fibrosis in mouse heart as determined by picrosirius red staining (Figure 1A). It is noteworthy that a single injection of ISP was not enough to induce enlargement of heart size or cardiac hypertrophy (left graph in Figure 1B). The quantification of the fibrosis area is shown in the graph on the right in Figure 1B.

In this simple experimental model, we first examined whether the PCAF protein amount was altered. Because we were interested in the roles of the individual cellular components in response to fibrotic stresses, we isolated cardiomyocytes and fibroblasts from the hearts after treatment with ISP. In the isolated cardiomyocytes, PCAF protein amounts were not altered by treatment with ISP (Figure 1C). In the isolated cardiac fibroblasts, however, PCAF protein amounts were reduced (Figure 1D). We measured the mRNA level of the *PCAF* by quantitative RT-PCR and again observed that the level was downregulated, whereas the mRNA levels of *Acta2* and *Col1a1*, both fibrosis markers, were increased (Figure 1E).

Many transcription factors work by binding to specific target DNA sequences or other proteins and thereby by modulating their functions. The PCAF is an epigenetic regulator, whose function is to “write” acetyl marks to its target proteins by inducing covalent bonds [30]. Thus, in addition to possessing the properties of a binding partner, like transcription factors do, these types of regulators work as enzymes to catalyze certain biochemical events such as acetylation [31]. Therefore, to delineate the function of the PCAF in fibroblasts, we should examine not only protein amounts or localization with respect to serving as a binding platform, but also enzymatic activity. We first measured the PCAF activity in cardiomyocytes isolated from ISP-administered mice, but observed no significant increase (Figure 1F). To our surprise, however, we found an increase, but not a decrease, in the PCAF activity in isolated cardiac fibroblasts (Figure 1G). Although the increase was about 2-fold, considering the PCAF amount was reduced to 70%, we estimated the overall increase in activity to be 2.8-fold.

### 2.2. TGF-β1 Reduces the PCAF Amount but Increases Activity in Human Cardiac Fibroblasts

**Figure 2:**
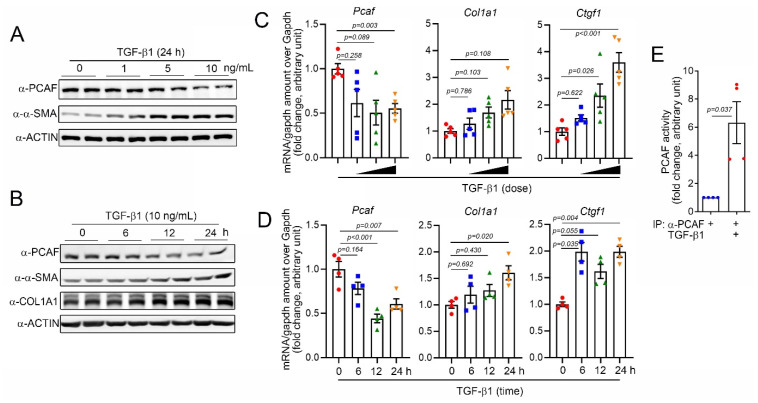
TGF-β1 reduced PCAF mRNA and protein but induced its acetylase activity. (**A**) TGF-β1, a fibrosis-inducing cytokine, decreased the PCAF protein amount in a time-dependent manner, whereas it increased α-SMA. (**B**) Dose-dependent effect of TGF-β1 on the PCAF protein reduction. (**C**) Changes in mRNA level determined by quantitative RT-PCR with TGF-β1 0–10 ng/mL used for 24 h. (**D**) Changes in mRNA level determined by quantitative RT-PCR with TGF-β1 (10 ng/mL) treated for 0–24 h. Both *COL1A1* and *CTGF* are fibrosis markers. (**E**) TGF-β1 significantly increased the PCAF acetylase activity.

For the mechanistic study, we next used primary human ventricular cardiac fibroblasts (NHCFs). TGF-β1 is well-known to induce the activation of fibroblasts by regulating the Smad signaling pathway [32]. Indeed, in our experimental models, TGF-β1 successfully increased the protein amounts of α-SMA and COL1A1, both fibrosis markers, in a time- and a dose-dependent manners (Figure 2A,B, respectively). The treatment with TGF-β1 also increased the mRNA levels of *COL1A1* and *CTGF* (Figure 2C,D). As observed in the in vivo condition (Figure 1), the PCAF protein amounts were significantly decreased in TGF-β1-treated cardiac fibroblasts in a time- and a dose-dependent fashions (Figure 2A,B, respectively). The mRNA levels of the *PCAF* were also decreased by TGF-β1 (Figure 2C,D). However, the treatment with 10 ng/mL TGF-β1 for 24 h significantly increased the PCAF activity (Figure 2E).

### 2.3. PCAF Is Required for TGF-β1-Mediated α-SMA and COL1A1 Induction 

α-SMA is an actin isoform of the vascular smooth muscle cellsand is used as a key hallmark of myofibroblasts. Since α-SMA is located mainly in the microfilament bundles, it is closely involved in regulating the contractility and mobility of cells responsible for healing wounds [33]. α-SMA is a direct target of TGF-β1 in the progression of fibrosis; during fibrosis, TGF-β1 induces α-SMA expression and its incorporation into stress fibers, which enhances the contractile properties of myofibroblasts [34].

**Figure 3:**
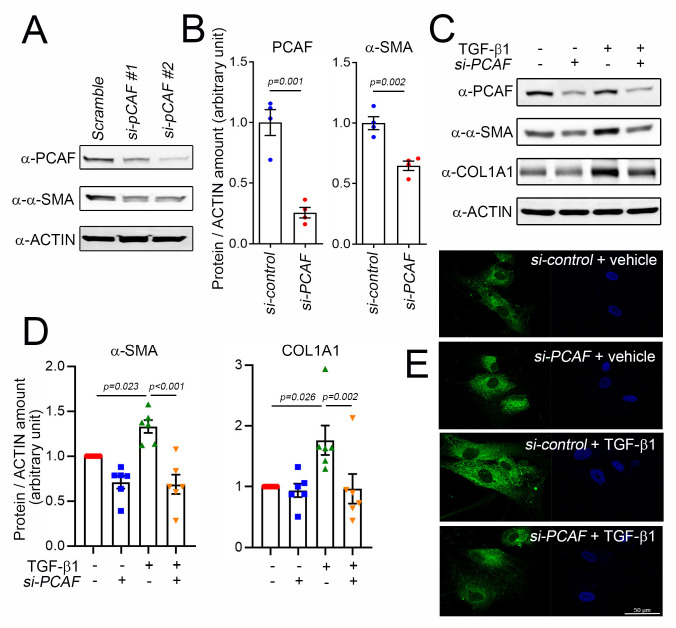
Knockdown of PCAF prevented TGF-β1-induced COL1A1 and α-SMA in human cardiac fibroblasts. (**A**) Efficiency of two independent sets of *PCAF siRNA*. For the following studies, *PCAF siRNA* #2 with better knockdown efficiency was used. (**B**) Treatment with *PCAF siRNA* #2 reduced the protein amounts of α-SMA and PCAF. (**C**) *PCAF siRNA* prevented the TGF-β1-induced increase in α-SMA and COL1A1. (**D**) Quantification results. (**E**) Immunofluorescent analysis to show COL1A1 in human cardiac fibroblasts. Note that the increase in the cytoplasmic abundance of the fluorescence (distribution of COL1A1, third panel) was significantly diminished by the simultaneous treatment with *PCAF siRNA* (fourth panel).

To investigate the role of the PCAF in α-SMA expression and cardiac fibrosis, we tried to check the effect of *PCAF siRNA* on the TGF-β1-mediated induction of α-SMA. Two different siRNAs were checked, and both successfully downregulated the protein amount of the PCAF (Figure 3A). The following study was performed with *PCAF siRNA* #2. The protein amount of α-SMA was significantly reduced by *PCAF siRNA* in the absence of TGF-β1 (Figure 3A,B). TGF-β1 significantly increased the α-SMA protein amount. However, the increase was completely blocked by *PCAF siRNA* (Figure 3C,D left graph). COL1A1 is a major structural protein associated with fibrosis [35]. COL1A1 was also increased by treatment with TGF-β1, which was blocked by *PCAF siRNA* (Figure 3C,D). TGF-β1 increased the immunofluorescent intensity of COL1A1 in the cytoplasm of human cardiac fibroblasts, which was then inhibited by *PCAF siRNA* (Figure 3E).

### 2.4. PCAF Regulates Cardiac Fibroblast Contraction, Migration, and Matrix Metalloproteinase (MMP) Activity

**Figure 4:**
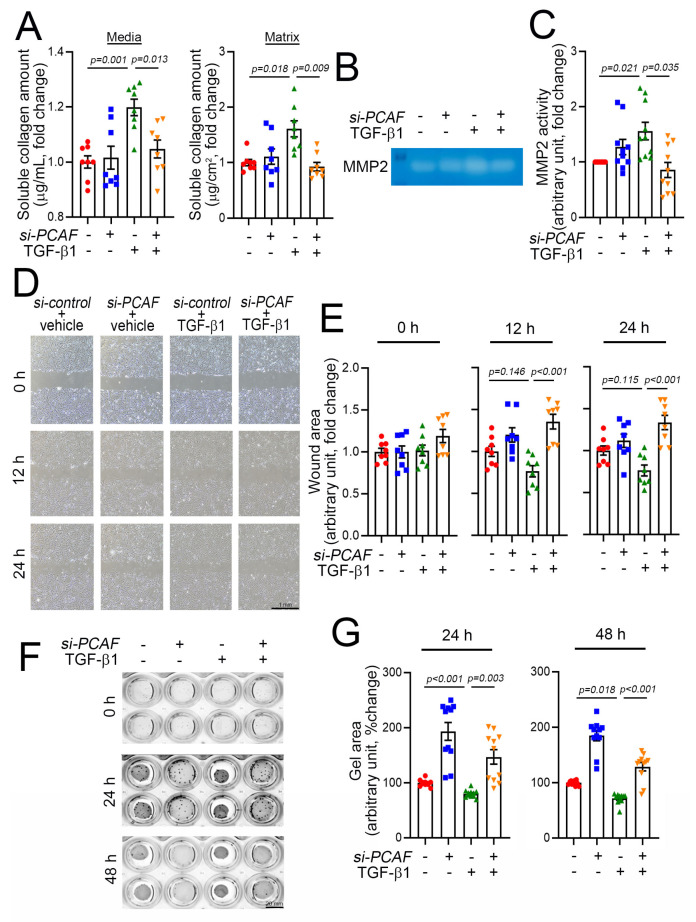
PCAF results in cardiac fibroblast contraction, migration, and MMP2 activity. (**A**) Changes in medial collagen in the culture media (**left**) and matrix collagen (**right**) on the well surface that were generated by human cardiac myofibroblasts. Notably, TGF-β1-induced increases in both medial and matrix soluble collagen amounts were abolished by *PCAF siRNA*. (**B**) MMP2 activity determined by gelatin zymography assay. (**C**) Quantification result of the gelatin zymography assay. (**D**) Wound healing assay to observe the fibroblast migration ability. The photographs were taken 0, 12, and 24 h after the scratch of the confluently cultured myofibroblasts. See the methods for detailed explanation. (**E**) Quantification results. The wound area (cell-free area) was measured by ImageJ software as described in the methods. TGF-β1 showed a tendency to reduce the wound area, which suggested increased migration (3rd bars and green dots at 12 h and 24 h). However, the migration was significantly inhibited by *PCAF siRNA* (4th bars and orange dots in 12 h and 24 h). (**F**) TGF-β1-induced gel contraction assay (3rd row in the wells), which was significantly attenuated by treatment with *PCAF siRNA* (4th row) in 24 h and 48 h. See the methods for explanation. (**G**) Quantification results.

Fibroblast activation is accompanied by an increase in synthesis of various types of collagen and the secretion of collagen into the ECM. The release of collagen is mediated by TGF-β1, and the released collagen works as a modulator of ECM rearrangement to increase the matrix compaction and thereby induce scar formation. Thus, the release of collagen into the extracellular components is one of the key features of fibroblast activation and thereby the initiation of fibrosis [36]. The released collagens can be measured by use of the Sircol soluble collagen assay kit as described previously [37]. As expected, using this method, we found that medial and matrix soluble collagen were increased in the culture media and on the well surface, respectively, when the cells were treated with TGF-β1 (Figure 4A). This increase in release, however, was completely blocked, when the cells were simultaneously treated with *PCAF siRNA* (Figure 4A).

The rearrangement of the ECM requires an increase in MMP activity, which influences the activation and proliferation of fibroblasts during fibrosis. This increase in MMP activity causes a “melting-down” of the ECM, which stimulates the fibroblasts to migrate into the wound area [38]. Many MMPs have been documented, and among these proteinases, MMP2 and MMP9 are key factors in inducing the degradation of gelatin, a partially hydrolyzed collagen [39]. MMP2 activity can be measured by the gelatin zymography assay. In our experimental model using acrylamide gel-containing gelatin, TGF-β1 increased MMP2 activity, which was blocked by simultaneous treatment with *PCAF siRNA* (Figure 4B,C).

Activated fibroblasts migrate to the injured area, and the migration capacity of the fibroblasts can be measured by using the conventional wound healing assay [40]. In this study, the results of the wound-healing assay showed that the treatment with TGF-β1 tended to reduce the cell-free wound area (Figure 4D and green triangular dots in Figure 4E). At 12 h and 24 h, however, the TGF-β1-mediated effects on cell migration were significantly attenuated by co-treatment with *PCAF siRNA* (Figure 4D and orange-colored triangular dots in Figure 4E).

The activation of fibroblasts results in the contraction of cells, which can be measured by the gel contraction assay [41]. The treatment with *PCAF siRNA* itself inhibited spontaneous gel contraction, as measured by relaxation of the gel area (second lines of wells in Figure 4F and blue dots in Figure 4G). TGF-β1 induced the contraction of the gel (third lines of wells in Figure 4F and green dots in Figure 4G). However, the contraction was completely abolished by *PCAF siRNA* (fourth lines of wells in Figure 4F and orange-colored dots in Figure 4G). These results suggest that the PCAF is required for the TGF-β1-mediated activation of fibroblasts.

### 2.5. PCAF Acetylates SMAD2 to Induce Its Phosphorylation and Nuclear Translocation

**Figure 5:**
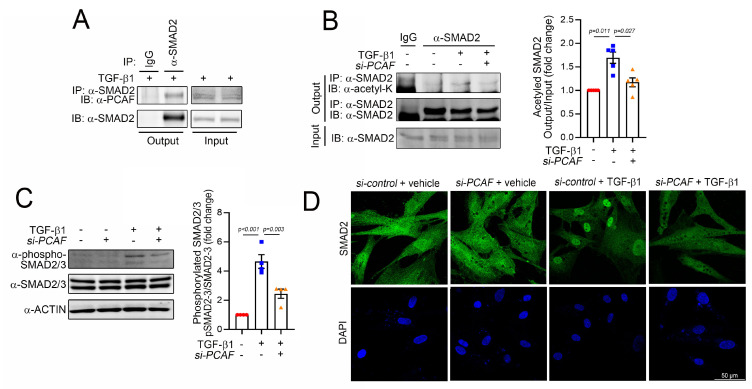
PCAF acetylates and activates SMAD2 in human cardiac fibroblasts. (**A**) Immunoprecipitation assay to show the interaction between SMAD2 and the PCAF. The endogenous SMAD2 precipitated the endogenous PCAF. (**B**) Immunoprecipitation-based acetylation assay. The TGF-β1-induced acetylation of SMAD2 was significantly attenuated by *PCAF siRNA*. Quantification results are shown in the graph on the right. (**C**) Phosphorylation of SMAD2/3 and its quantification results. (**D**) Fluorescent immunocytochemistry for SMAD2. TGF-β1 induced the nuclear localization of SMAD2, which was blocked by *PCAF siRNA*.

Most signals to induce fibrosis merge into the SMAD signaling pathways, and SMAD family proteins have diverse functions, depending on their specific ligands, such as bone morphogenetic proteins (BMPs) and TGF-βs [14]. For example, SMAD2/3 works as a profibrotic mediator of TGF-βs, while SMAD1/5/(8)9, primarily activated by BMPs, functions to prevent fibrosis [42]. Among these SMAD proteins, SMAD2 is a key protein for fibrosis. Thus, we postulated that PCAF may directly target SMAD2 to regulate fibrosis. To test this possibility, we first checked whether these two proteins associate directly with each other. The immunoprecipitation assay showed that endogenous SMAD2 successfully precipitated the PCAF in NHCFs in the presence of TGF-β1 (Figure 5A).

Like many histone acetyltransferases and histone deacetylases, the PCAF has multiple target proteins other than histones. For example, p53 and peroxisome proliferator-activated receptor gamma coactivator 1-alpha are well-known nonhistone targets of the PCAF [31,43]. Indeed, our laboratory also found that even HDAC2 is a nonhistone target of the PCAF and that PCAF-mediated acetylation of HDAC2 causes its activation [24]. Thus, we postulated that SMAD2 might be a target of the PCAF and thereby that the acetylation of SMAD2 could play a role in fibrosis. We first checked the acetylation of SMAD2 by immunoprecipitation-based assay with an acetyl lysine antibody. The treatment with TGF-β1 increased the acetylation of SMAD2 (3rd lane of left gels in Figure 5B). The acetylation, however, was blocked by *PCAF siRNA* (4th lane). The changes in acetylation were quantified (right bar graph in Figure 5B). These results suggest that SMAD2 acetylation is required for TGF-β1-mediated fibroblast activation and that the PCAF is at least in part involved in the acetylation.

To carry out their transcriptional activities, SMAD2/3 should translocate into the nucleus and reside on their target promoter region [44]. Their translocation can be achieved by the phosphorylation of the specific serine residues at 465/467 in SMAD2 and 423/425 in SMAD3, which results in the binding of SMAD4 [45]. Thus, the phosphorylation of these residues is the final critical step for the activation of SMAD signaling. We checked whether the PCAF affected this final step of SMAD2 phosphorylation and localization. As expected, TGF-β1 successfully increased the phosphorylation of SMAD2/3 (3rd lane of the left gel and 2nd bar of the right graph in Figure 5C). However, this increase was significantly inhibited by the treatment with *PCAF siRNA* (4th lane of the left gel and 3rd bar of the right graph in Figure 5C). The treatment with TGF-β1 dramatically induced the localization of SMAD2 into the nucleus (2nd images in Figure 5D). In the presence of *PCAF siRNA*, however, TGF-β1 failed to do so (4th images in Figure 5D). These results suggest that the PCAF is needed for the TGF-β1-mediated phosphorylation and translocation of SMAD2 during fibroblast activation.

## 3. Discussion

In the present study, we elucidated that the PCAF is required for the activation of cardiac fibroblasts in response to TGF-β1. In this signaling pathway, the PCAF binds directly to SMAD2 and induces its acetylation. Interference with PCAF signaling has the effect of reducing SMAD2 phosphorylation and thereby nuclear localization, which suggests that the PCAF is required for the eventual activation of SMAD2 in cardiac fibroblasts.

Multiple cellular processes merge through a relatively small number of intracellular signaling pathways that share secondary messengers. One example of the shared pathways is the TGF-β pathways. Indeed, TGF-β is a pleiotropic cytokine, and many biological events, such as cellular development, growth, proliferation, and death, are tightly regulated by TGF-β and its associated signaling [46]. The TGF-β ligand initiates the signaling by binding to the serine/threonine kinase TGF-β receptors [47], which then causes the phosphorylation of SMAD2/3 and hetero-oligomerization of SMAD4 [44]. These events induce the translocalization of the complex into the nucleus to initiate the transcription of target genes [48]. One of the main effects of SMAD signaling is to induce fibrosis in fibroblasts [14] or in other cell types via a specialized mechanism known as the EMT or EndMT [11,12]. Because of the complexity and cell-type specificity of these SMAD-related pathways, however, a better understanding is needed in specific cell types in cardiac fibrosis. Indeed, the role of SMAD acetylation and its acetyltransferase in association with cardiac fibrosis has not been reported.

Diverse PTMs of proteins cause significant conformational changes to the proteins. Besides phosphorylation, SMAD2/3 undergoes acetylation, which results in an increase in its binding to target DNA to initiate transcription. In addition to histones, p300/CBP, a well-known histone acetyltransferase [49], catalyzes SMAD2/3 as a nonhistone target, which then results in an increase in transcription [28,50]. Lysine 19, 20, and 39 on SMAD2 are known to be p300 target residues [28,49]. Indeed, as a therapeutic approach to reversing fibrosis, interference with SMAD2 acetylation either by the DAXX transcription factor, as shown in hepatocytes [51], or by the p300/CBP inhibitor garcinol, as shown in a nondiabetic renal fibrosis model [26], can be considered. Using a less specific histone acetyltransferase inhibitor, curcumin, in a diabetic cardiac fibrosis model, Bugyei-Twum et al. [52] also reported that p300 is associated with SMAD2 acetylation in H9C2 cells.

It is noteworthy that acetyltransferase can be counteracted by deacetylase. Indeed, Sirtuin1 and Sirtuin6 have been elucidated as SMAD2/3 deacetylases. Sirtuin1 is shown to reverse SMAD3 acetylation in rat renal NRK49F fibroblasts [53] and to prevent EndMT during cardiac fibrosis [54]. Sirtuin6 is shown to deacetylate Lys54 on Smad2 to prevent liver fibrosis [55], which suggests that like acetylation, the deacetylation of SMAD2/3 varies, depending on the cell types or related disease models.

The PCAF also has been reported to induce the acetylation of SMAD2/3 in HEK293T cellular models. For example, the PCAF is shown to directly interact with SMAD2/3 and potentiate its transcriptional activity [27,28]. The same research group also found that lysine 19 is responsible for PCAF-mediated acetylation [28]. Although we did not specify the exact lysine residue, in the present work, we also found that the PCAF directly bound to SMAD2 and induced its acetylation in human cardiac fibroblasts, which suggests a common signaling pathway. We also found that the PCAF is required for the phosphorylation of SMAD2 and its translocation. It is not clear, however, how acetylation of SMAD2 affects phosphorylation or vice versa. It is also not known whether these two post-translational modifications, i.e., acetylation for DNA binding and phosphorylation for intracellular localization, are independent.

Previously, we clearly reported that the PCAF causes cardiac hypertrophy by inducing the acetylation of HDAC2 K74 [24]. The acetylation of HDAC2 is required for the sequential phosphorylation of HDAC2 S394, a hypertrophy-responsive phosphorylation residue of HDAC2, which then activates HDAC2 for the initiation of cardiac hypertrophy [20,21]. In the current work, we first showed that the PCAF is necessary for cardiac fibrosis by activation of the SMAD signaling pathways. Thus, together with our previous report describing the prohypertrophic function of the PCAF in cardiomyocytes [24], our findings lead us to conclude that the PCAF plays dual roles to induce cardiac remodeling and that the modulation of PCAF signaling may provide a therapeutic platform to overcome adverse cardiac remodeling following cardiovascular diseases.

## 4. Materials and Methods

### 4.1. Antibodies and Reagents

Isoproterenol (ISP) hydrochloride, bovine serum albumin (BSA), taurine, 2,3-butanedione monoxime, 2,2,2-tribromoethanol, and 2-methyl-2-butanol were purchased from Sigma (St. Louis, MO, USA). Collagenase type B was from Hoffmann-La Roche (Basel, Switzerland). Hyaluronidase was from Worthington Biochemical (Lakewood, NJ, USA).

Antibodies used were as follows: anti-PCAF (#3378S), anti-SMAD2 (#5339S), anti-SMAD2/3 (#8685S), and phospho-SMAD2/3 (#8828S) were from Cell Signaling Technology (CST, Danvers, MA, USA); anti-actin (#A2066) that recognized the pan-actin was from Thermo Fisher Scientific (Thermo Fisher Scientific, Waltham, MA, USA); anti-alpha-smooth muscle actin (α-SMA) (#ab5694), collagen type I alpha 1 (COL1A1) (#ab34710), and acetyl-lysin (#ab21623) were from Abcam (Cambridge, UK); β-actin (#sc-47778), normal mouse IgG (#sc-2025), and normal rabbit IgG (#sc-2027) were from Santa Cruz (Santa Cruz Biotechnology, Dallas, TX, USA); horseradish peroxidase (HRP)-conjugated secondary antibody against mouse IgG or rabbit IgG was from Cell Signaling Technology (Danvers, MA, USA); Alexa Fluor 488-conjugated anti-mouse IgG (#A110001) or rabbit IgG (#A110008) was from Thermo Fisher Scientific (Thermo Fisher Scientific, Waltham, MA, USA).

### 4.2. Isoproterenol Administration

Male C57BL/6J mice were purchased from Damul Science (Damul Science, Daejeon, Korea). Eight-week-old male mice were injected with 160 mg/kg ISP in PBS or the equivalent volume of PBS by a single i.p. injection as described in Forte et al. [29]. At 1 week after injection, the mice were sacrificed by carbon dioxide inhalation, and heart tissues were harvested.

### 4.3. Isolation of Mouse Cardiac Fibroblasts and Myocytes

Mouse cardiac fibroblasts and myocytes were isolated by using a Langendorff perfusion system as previously described [56]. Briefly, the mice were anesthetized with 1.2% 2,2,2-tribromoethanol, and hearts were dissected. The hearts were washed with Ca^2+^-free Tyrode buffer (10 mmol HEPES pH 7.4, 137 mmol NaCl, 5.4 mmol KCl, 1 mmol MgCl_2_, 10 mmol glucose, 5 mmol taurine, and 10 mmol 2,3-butanedione monoxime) for 5 min followed by perfusion with Ca^2+^-free Tyrode buffer containing hyaluronidase (0.1 mg/mL) and collagenase type B (0.35 U/mL) for 15 min. Perfused hearts were dissociated into small pieces with forceps and suspended in Tyrode buffer containing 5% BSA to stop enzyme activity. The cells were passed through a 100 µm cell strainer (#352360, Falcon, NY, USA), and myocytes were allowed to settle by gravity for 10 min. The supernatants were briefly centrifugated to enrich cardiac fibroblasts.

### 4.4. Histologic Analysis

Mouse hearts were isolated and fixed in 4% paraformaldehyde at 4 °C for 24 h. Fixed hearts were embedded in paraffin and cross-sectioned. Picrosirius red staining (#ab150681, Abcam) was carried out to determine fibrosis, following the manufacturer’s instruction. The area of fibrosis was measured by use of ImageJ software.

### 4.5. Cell Culture and Differentiation

NHCFs were purchased from LONZA (#CC-2904, Lonza, Basel, Switzerland) and maintained in a fibroblast basal medium (FBM, #CC-3131, Lonza, Basel, Switzerland) supplemented with an FGM-3 Bullet Kit (#CC-4526, Lonza, Basel, Switzerland) at 37 °C in a humidified incubator with 5% CO_2_. NHCFs between passages 6 and 9 were used for all experiments. To induce myofibroblast differentiation, NHCFs were starved in FBM for 12 h followed by treatment with human TGF-β1 (#PHG9214, Gibco, MA, USA).

### 4.6. Small Interfering RNA and Transfection

Predesigned small interfering RNA (siRNA) for the PCAF (#8805) and AccuTarget negative control siRNA (#SN-1003) were purchased from Bioneer (Daejeon, Korea). siRNA transfections were performed using Lipofectamine RNAiMax Transfection Reagent (#13778075, Thermo Fisher Scientific, Waltham, MA, USA), according to the manufacturer’s protocol.

### 4.7. RNA Extraction and Quantitative Real-Time PCR

Total RNA was isolated using TRI reagent (#TR118, Molecular Research Center, OH, USA), according to the manufacturer’s protocol. DNase I (#2270A, Tokyo, Takara) was used to remove residual DNA. Complementary DNA (cDNA) was synthesized using RevertAid reverse transcriptase (#EP0442, Thermo Scientific) and random hexamers (#SO142, Thermo Scientific). cDNAs were used to perform quantitative real-time PCR by using a QuantiTect SYBR Green PCR kit (#204143, Qiagen, Germantown, MD, USA) and a Rotor-Gene Q real-time PCR cycler (Qiagen). The primer sequences are listed in Table 1.

### 4.8. Western Blot Analysis and Immunoprecipitation

NHCFs were lysed using a radioimmunoprecipitation assay lysis buffer (RIPA, #R2002, Biosesang, Seongnam, Korea). The protein concentration in cell lysates was determined by use of bicinchoninic acid protein assay kits (#23225, Thermo Fisher Scientific). The lysates were separated by SDS-polyacrylamide gel and then transferred to PVDF membranes (#IPVH00010, Merck Millipore, Darmstadt, Germany). The membranes were blocked with 5% skim milk (#232100, BD, New Jersey, USA) followed by incubation with primary antibody at 4 °C. The membranes were incubated with a horseradish peroxidase-conjugated secondary antibody. Immobilon chemiluminescent HRP substrates (#P90720, Merck Millipore) and FUSION-FX-SPECTRA (Vilber GmbH, Eberhardzell, Germany) were used to develop bands on the blot. Band intensities were analyzed using ImageJ software.

For immunoprecipitation, 1–2 mg of cell lysates was precleared by protein A/G PLUS-Agarose (sc2003, Santa Cruz Biotechnology) at 4 °C for 1 h prior to incubation with an antibody. A primary antibody was added to precleared lysates with gentle shaking at 4 °C. Protein A/G PLUS-Agarose (#sc-2003, Santa Cruz Biotechnology) was used to enrich immunocomplex for 4 h. The bead-based immunocomplex was retrieved by centrifugation at 3000 rpm for 3 min and was washed twice with the lysis buffer. Bead-pellets were mixed with a 2× NuPAGE SDS sample buffer (#LC2676, Invitrogen, Waltham, MA, USA) with beta-mercaptoethanol and subjected to boiling. The corresponding purified IgG was used as a negative control.

### 4.9. PCAF Activity Assay

PCAF activity was measured by a HAT assay kit (#ab65352, Abcam), according to the manufacturer’s instructions. Briefly, NHCFs or hearts were lysed with a RIPA buffer. Lysates were incubated with the PCAF antibody and the protein A/G PLUS-Agarose (#sc2003, Santa Cruz Biotechnology). The lysates were centrifugated and washed twice with the lysis buffer. Then, the bead-based immunocomplex was subjected to HAT assay substrates at 37 °C for 4 h. Absorbances were measured at 440 nm using a Spectramax Abs Plus (Molecular Devices, San Jose, CA, USA). The corresponding IgG was used as a blank and subtracted from each experimental sample.

### 4.10. Sircol Collagen Assay

The contents of matrix collagen and soluble collagen secreted into the culture media were determined by the use of the Sircol soluble collagen assay kit (#S1000, Biocolor Assays, Ireland), following the manufacturer’s instructions. To extract matrix collagen, the cells were digested with 0.5 M acetic acid containing 0.1 mg/mL pepsin (P7012, Sigma-Aldrich, St. Louis, MO, USA) at 4 °C for 24 h. Then, acid extracts were neutralized by an acid-neutralizing reagent. To measure soluble collagen content, conditioned media and acid extracts were incubated with a Sircol dye reagent at room temperature for 30 min on an orbital shaker. The dye complex was centrifugated at 12,000 rpm for 10 min, and the dye-bound pellets were dissolved in an alkali reagent. The absorbance values were recorded at 555 nm using a Spectramax Abs Plus (Molecular Devices, San Jose, CA, USA). Total collagen contents were calculated from a standard curve with provided collagen.

### 4.11. Gelatin Zymography Assay

A conditioned medium was used to measure matrix metalloproteinase 2 (MMP2) activity. The media were collected and subjected to ultra centrifugal filters (UFC503008, Millipore, Burlington, MA, USA) to concentrate proteins. Fifteen micrograms of the conditioned media proteins were separated by 7.5% SDS-polyacrylamide gel containing 1 mg/mL gelatin under nonreducing conditions. To remove SDS, the gels were washed twice for 30 min with a wash buffer (2.5% Triton X-100, 50 mM Tris HCl, 5 mM CaCl_2_, and 1 μM ZnCl_2_) followed by incubation with the reaction buffer (1% Triton X-100, 50 mM Tris HCl, 5 mM CaCl_2_, and 1 μM ZnCl_2_) at 37 °C for 24 h. To visualize bands, the gels were stained with a DirectBlue Gel Staining Solution (#BDS-1000, BIOMAX, Seoul, Korea) and destained with 10% acetic acid and 50% methanol. Clear bands on the gel were quantified using ImageJ software.

### 4.12. Wound Healing Assay

The ability of the cells to migrate was evaluated by a scratch wound healing assay. When NHCFs reached confluence, the complete medium was changed to serum-free medium to block proliferation, and cells were treated with siRNA for 12 h. To create a scratch, the cell layer was scratched with a sterile micropipette tip and washed with PBS twice. Then, the cells were incubated with serum-free media with TGF-β1 for 24 h. The wound area was recorded using an Eclipse Ts2R microscope (Nikon, Tokyo, Japan) and calculated using ImageJ software.

### 4.13. Collagen Gel Contraction Assay

The contractile activity of NHCFs was measured by collagen gel contraction assay. After the knockdown, 2 × 10^5^ cells were resuspended with serum-free media containing 1 mg/mL Collagen type I (#354231, Corning Life Sciences, NY, USA). The collagen–cell mixture was mixed with 1M NaOH and immediately transferred to the wells of a 12-well plate. The gels were allowed to polymerize at room temperature for 30 min and gently detached with a sterilized spatula. To initiate contraction, the gels were covered with serum-free media containing TGF-β1. The gels were imaged at 0, 24, and 48 h by use of the ENDURO GDS gel documentation system (Labnet International, Iselin, NJ, USA), and gel size was calculated by using ImageJ software.

### 4.14. Immunocytochemistry

NHCFs were grown in chamber slides (#154534, Thermo Fisher Scientific) and treated with TGF-β1 after silencing the PCAF. The NHCFs were fixed with 3.7% (*v*/*v*) paraformaldehyde and permeabilized with 0.2% Triton X-100 in PBS. After blocking with 5% BSA at room temperature for 30 min, the cells were incubated with primary antibodies against SMAD2 (1:100; CST) or COL1A1 (1:100; Abcam) in a permeabilization buffer (0.2% Triton X-100, 1% BSA in PBS). Alexa-conjugated secondary antibodies (Thermo Fisher Scientific) were used to confirm primary antibodies. Cells were covered with antifade solution containing 6-diamidion-2-phenylindole (DAPI, #S36939, Thermo Fisher Scientific). The cells were visualized by use of a Zeiss LSM 710 confocal microscope (Carl Zeiss, Oberkochen, Germany).

### 4.15. Statistics

Statistical analysis was performed using PASW Statistics 26 (SPSS, IBM Corp, Chicago, IL, USA). Data are presented as means ± standard error of the mean (SEM). To compare two independent groups, we used a two-tailed unpaired Student’s t-test or the nonparametric Mann–Whitney U test after checking for a normal distribution. To compare more than two groups, we used one-way analysis of variance (ANOVA) or two-way ANOVA with post hoc tests according to the levels of independent variables. When the interaction between independent variables was significant, stratification was performed for a pairwise comparison. The assumption of equal variance was confirmed using Levene’s test. For post hoc tests, we performed Tukey’s honestly significant difference (HSD) for multiple comparisons in equal variance, whereas the Dunnett’s T3 test was used for unequal variance. Statistical significance was considered when the *p*-value was <0.05.

## Figures and Tables

**Table 1:** Real-time PCR primer sequences used in this study.

**Human**		
**Gene**	**Forward (5′ to 3′)**	**Reverse (5′ to 3′)**
*PCAF*	GAAGAGAACAGAAGCTCCAGG	GCAATTGGTAAAGACTCGCTG
*ACTA2*	CCATCATGCGTCTGGATCTG	ACGCTCAGCAGTAGTAACGA
*COL1A1*	TCTGCAACATGGAGACTGGT	TCGAACTGGAATCCATCGGT
*CTGF*	ATTAGAGCCAACTGCCTGGT	AGGAGGCGTTGTCATTGGTA
*FN1*	GGTACAGGGTGACCTACTCG	GGAATAGCTGTGGACTGGGT
*GAPDH*	GTCGGAGTCAACGGATTTGG	TGACGGTGCCATGGAATTTG
**Mouse**		
**Gene**	**Forward (5′ to 3′)**	**Reverse (5′ to 3′)**
*Pcaf*	GCCGTGTCATTGGTGGTATC	GGGTTCCATAGCCCTTGACT
*Acta2*	CTCCCTGGAGAAGAGCTACG	CGCTGACTCCATCCCAATGA
*Col1a1*	TCCCTGGAATGAAGGGACAC	CTCTCCCTTAGGACCAGCAG
*Ctgf*	AGTGTGCACTGCCAAAGATG	CCAGGCAAGTGCATTGGTAT
*Fn1*	ACCCTTGGCCTCCAAGTATC	CAGAGGCTGCAGGGTAGTAA
*Tgfb1*	TTGCTTCAGCTCCACAGAGA	CAGAAGTTGGCATGGTAGCC
*Gapdh*	GCATGGCCTTCCGTGTTCCT	CCCTGTTGCTGTAGCCGTATTCAT

Abbreviations: PCAF, p300/CBP-associated factor; ACTA2, alpha-smooth muscle actin; COL1A1, collagen type I alpha 1; CTGF, connective tissue growth factor; FN1, fibronectin 1; GAPDH, glyceraldehyde-3-phosphate dehydrogenase; Tgfb1, transforming growth factor beta1.

## Abbreviations

α-SMA	alpha-smooth muscle actin
COL1A1	collagen type I alpha 1
CTGF	connective tissue growth factor
ECM	extracellular matrix
EndMT	endothelial-to-mesenchymal transition
EMT	epithelial-to-mesenchymal transition
FN1	fibronectin 1
GAPDH	glyceraldehyde-3-phosphate dehydrogenase
HDAC2	histone deacetylase 2
HDAC5	histone deacetylase 5
HRP	horseradish peroxidase
ISP	isoproterenol
MMP	matrix metalloproteinase
PCAF	p300/CBP-associated factor
SMAD	small mothers against decapentaplegic
TGF-β	transforming growth factor-β
